# Kinesin light chain 1 interacts with NS1 and is a susceptibility factor for dengue virus infection in mosquito cells

**DOI:** 10.1099/jgv.0.002132

**Published:** 2025-07-16

**Authors:** Juan Manuel Castillo, Raymundo Cruz-Pérez, Daniel Talamás-Lara, Juan E. Ludert

**Affiliations:** 1 Department of Infectomics and Molecular Pathogenesis, Center for Research and Advanced Studies (Cinvestav), Mexico City, Mexico; 2 Electron Microscopy Unit (LANSE), Center for Research and Advanced Studies (Cinvestav), Mexico City, Mexico

**Keywords:** dengue, dengue virus, kinesin light chain 1, lipid droplets, protein NS1

## Abstract

A hallmark of the dengue virus (DENV) infection is the manipulation of host cell membranes, lipid trafficking and lipid droplets (LD), all cellular functions that depend on the cytoskeleton and the cytoplasmic streaming system. We previously reported the interaction between the DENV non-structural (NS1) protein and members of the kinesin motor complex in the *Aedes albopictus* cell line C6/36. In this work, we present evidence indicating that the protein kinesin light chain 1 (KLC1) is indeed a susceptibility factor for the DENV replicative cycle in mosquito cells. The interaction between NS1 and KLC1 was confirmed by proximity ligation and co-immunoprecipitation assays in cells harvested 24 hpi. In addition, transmission immunoelectron microscopy showed KLC1 decorating the surface of vacuoles in association with NS1. Increased levels of KLC1 were observed starting at 6 hpi, suggesting that virus infection stimulates KLC1 synthesis. Silencing KLC1 expression results in a reduction in viral genome synthesis, decreased secretion of NS1 and a reduction of virus progeny by nearly 1 log. In agreement, similar affectations were observed in infected cells transfected with a peptide that competes and interferes with the interaction between KLC1 and its cargo molecules. Of note, both silencing the expression and interfering with the function of KLC1 resulted in a disorganization of LD, which decreased in number and increased in area, in mock or infected cells. These results, taken together, suggest that KLC1 is a host susceptibility factor for DENV in mosquito cells and appears to play an important role in the proper transport and homeostasis of LD required for flavivirus replication. However, modest colocalization was observed between NS1 and LD, and the significance of the KLC1 and NS1 interactions needs to be further investigated.

## Introduction

The genus *Orthoflavivirus* represents a group of vector-borne viruses that have become a continuous threat to the world’s public health, with the potential to cause major outbreaks [1]. Orthoflaviviruses like yellow fever virus, Japanese encephalitis virus and West Nile virus are a major public health concern in their respective subtropical areas [2]. In the past decade, the Zika virus raised an epidemiological alert in the Latin America and the Caribbean regions, and nearly half of the earth’s population lives in dengue virus (DENV) risk areas [3]. The highly mutagenic genome and the capacity of their vectors to adapt and take advantage of the world’s environmental shift make Orthoflaviviruses highly successful pathogens [4,6].

DENV is an icosahedral enveloped virus [7] with a +ssRNA genome of ~10 Kb in size that encodes for a polyprotein that is processed into three structural proteins (C, M and E) and seven non-structural proteins (NS1, NS2A, NS2B, NS3, NS4A, NS4B and NS5) [8]. In humans, after virus mosquito inoculation, the virus first infects skin Langerhans dendritic cells, then spreads to lymph nodes, peripheral blood, liver and spleen [9,10]. In *Aedes aegypti* and *Aedes albopictus* mosquitoes, the first tissue to get infected after blood feeding is the midgut; the infection then spreads to the haemocoel and, finally, the salivary glands [11].

A key feature in the *Orthoflavivirus* replication cycle is the manipulation of internal host membranes to form the replication organelles (RO) in endoplasmic reticulum (ER) derived membranes. Extensive remodelling of the ER membranes has been reported in both DENV and other closely related *Orthoflavivirus*-infected mammalian and mosquito cells [12,16]. It has been proposed that these intricate structures not only serve as scaffolds, where viral replication and assembly occur, but they also favour enzymatic retention, substrate accumulation and immune evasion in favour of viral replication [17,19]. Therefore, several DENV proteins have acquired the capacity to interact with cellular membranes [14,20]; for example, NS4A, which is thought to induce membrane curvature, anchoring itself in the cytosolic side of the RO via its transmembrane region [21], or NS1, which has been proposed to insert into the luminal side of membranes via its *β*-roll domain, also inducing membrane curvature and serving as a scaffold for the replication complexes (RC). Others, like NS3, have been reported to redistribute and stimulate the fatty acid synthase to viral RC to aid virus replication [22].

In addition, lipid hijacking is a well-recognized phenomenon in DENV-infected cells [23,24]. By altering lipid homeostasis and mobilization, Orthoflaviviruses obtain the building blocks necessary to establish viral RC [14] and assembly of nascent viral particles [22,25]. Namely, lipid droplets (LD), the organelles responsible for the storage and transport of neutral lipids that allow the cell to have a reservoir of energy and structural building blocks for survival [26], are targeted during *Orthoflavivirus* infection. Recent evidence suggests that in mosquitoes, LD accumulate in the midgut cells upon DENV infection and play a role in the insect immune response to infection [26], and the C protein of several Orthoflaviviruses is known to interact with the surface of LD for nucleocapsid formation [27,28]. Thus, targeting LD has been proposed as a potential strategy for the development of *Orthoflavivirus* antivirals [29].

The kinesin motor complex (KMC) is a system involved in the cytoplasmic streaming of cargos through the microtubules in a highly coordinated and active pattern [30,31]. It is composed of two heavy chains that perform the motor function and two light chains that serve as an adaptor that links the heavy chains with the cargo. Based on the subunits, the KMC can adopt several configurations, and this depends on the cell type and the cargo to be transported. The kinesin light chain 1 (KLC1), one of the subunits of the KMC, is composed of three domains; the N-terminal domain connects with the heavy chains [32], the tetratricopeptide repeat (TPR) domain is the region that interacts with cargo proteins [33] and the C-terminal domain has been proposed to attach to membranes [34]. Of note, the KMC is a system strongly related to LD motility [30], and there is evidence suggesting that KLC1 plays a significant role in the homeostasis of these organelles [35]. Conversely, KLC1 and other members of the KMC have been reported as targets of viral effectors to favour viral replication [36,38], likely due to their ability to traverse all the cells and be involved in a myriad of cell processes. Recently, KLC1 was identified as part of the interactome of DENV NS1 in mosquito C6/36 cells [39]. Here, we present evidence indicating that KLC1 certainly interacts with NS1 and is a susceptibility factor for DENV in C6/36 cells, involved in the mobilization of neutral lipids via LD. The results shed light on the mosquito cell–DENV interactions and identified potential new targets for antiviral strategies.

## Methods

### Cell cultures and viruses

The C6/36 cell line (CRL-1660; ATCC) was cultured with minimum essential medium (MEM) (30-2003; ATCC) with 5% FBS and 100 U ml^−1^ penicillin–streptomycin (Gibco, 15140122) at 27 °C and 5% of CO_2_. Confluent cell monolayers, grown in six-well plates, were infected with DENV-2, strain New Guinea, at an m.o.i.=3 in FBS-free MEM, for 2 h. Infections were allowed to proceed for 24 hpi, after replenishing the monolayers with MEM supplemented with FBS at 5% and antibiotics. BHK cells (BHK-21; ATCC CCL-10) were cultured in MEM with 5% of FBS and 100 U ml^−1^ penicillin–streptomycin at 37 °C and 5% of CO_2_.

### Cell viability

Cell viability was measured 24 h post-treatment with the MTS proliferation assay (Cell Titer 96 Aqueous; Promega, G3580) in 96-well plates, following the manufacturer’s recommendations.

### Gene silencing

Specific KLC1 siRNA duplexes were purchased from Integrated DNA Technologies and were targeted against the heptad repeat encoding region of *A. albopictus* KLC1 (>AALC636_019471.R27282). To carry out the silencing assays, C6/36 cell monolayers at 80% confluence were transfected with 200 nM of KLC1 siRNA duplex using 3 µl of lipofectamine 2000 (Invitrogen, 1168019), following the manufacturer’s instructions, and cells were processed for analysis 24 hours post transfection (hpt). AllStars Negative Control siRNAs were used as a control in the same concentration as the treatment (Qiagen, SI03650318). In the case of infected cells, siRNA transfection was done 2 h post-infection.

### Plaque formation

For progeny virus titration, cell supernatants were serially diluted (10-fold), and 0.2 ml of each dilution was added in triplicate onto confluent monolayers of BHK-21 cells grown in six-well plates in FBS-free MEM for 2 h at 37 °C. Then, 1 ml of overlay media (2× MEM mixed 1:1 with carboxymethyl cellulose at 1.8% in Milli-Q^®^ water) was added, and the cells were incubated for 5 days at 37 °C and 5% CO_2_. Next, the carboxymethyl cellulose was removed, and 500 µl of naphthol blue black (Sigma-Aldrich, N9002) was added for 24 h at room temperature. Finally, naphthol blue black was removed, monolayers were washed three times with tap water and the plaques were counted. Results were expressed as p.f.u. ml^−1^.

### Indirect immunofluorescence

At the appropriate times post-infection, culture medium was removed, and monolayers were fixed with 300 µl of a 4% paraformaldehyde solution for 30 min at 4 °C. Then, cells were washed with 0.2% PBS-Tween 20 and permeabilized with 300 µl of a 0.2% PBS-Triton X-100 solution for 10 min at room temperature. Afterwards, the glass slides were transferred to a humid chamber over a sheet of Parafilm^®^, and 50 µl of blocking solution (Glycine 25 mg ml^−1^, 1% FBS and 1% fish gelatin) was added for 2 h at 37 °C. The blocking solution was removed, and 50 µl of the solution with primary antibodies was added overnight at 4 °C. The solution with primary antibodies was removed, and the slides were washed three times for 10 min with PBS 0.2%-Tween 20, then 5 µl of a solution with secondary antibodies was added for 2 h at 37 °C. Finally, the solution with secondary antibodies was removed; a solution with DAPI (Sigma, D9542) 1:1,000 was added for 10 min, and the slides were washed three times for 10 min with 0.2% PBS-Tween 20 and once for 5 min with Milli-Q water. The slides were mounted with 5 µl of VectaShield (Vector, H-1000) and analysed on an LSM 900 confocal microscope complemented with an Airyscan 2 module. For protein colocalization, ~100 cells were counted, and the average colocalization index (Pearson correlation coefficient, PCC) was calculated with the Zeiss and Icy software.

Primary antibodies used in this study were rabbit hyperimmune serum anti-NS1 (produced in-house) and commercial mouse monoclonal anti-NS3 (GT2811), mouse monoclonal anti-NS4A (GTX132069), rabbit polyclonal anti-tubulin (GTX11307) and rabbit polyclonal anti-KLC1 (ABclonal, A5552). Secondary antibodies were anti-rabbit (Invitrogen, A21245) and anti-mouse (ABCam, AB150109) Alexa conjugated antibodies.

### Proximity ligation assays (PLA)

PLA were performed with a Duolink kit (Sigma-Aldrich, DUO92004) according to the manufacturer’s protocol. Briefly, infected cells were fixed at 24 hpi with a 4% paraformaldehyde solution for 30 min at 4 °C. Then, the slides were transferred to a humidity chamber, and one drop of the blocking solution was added for 1 h at 37 °C. Primary antibodies were added in a 1:100 dilution and incubated overnight at 4 °C. Following this, primary antibodies were removed, and the slides were washed with reagent A two times for 5 min each, followed by the addition of the minus and plus probes in a 1:5 dilution and incubated for 1 h at 37 °C. After incubation, the probe solution was removed, and the slides were washed two times for 5 min each with reagent A; then, the ligase was added in a 1:40 dilution for 30 min at 37 °C. After this, the ligase solution was removed, and the slides were washed two times for 5 min each with reagent A. Afterwards, the ligase solution was removed, and the polymerase was added in a 1:80 dilution for 100 min at 37 °C. Finally, the polymerase solution was removed, and the slides were washed two times for 10 min each with reagent B, then one time with reagent B 0.01X for 1 min. Negative technical controls included mock cells exposed to both primary antibodies and infected cells exposed only to KLC1 antibody. Cells were analysed under a confocal microscope, Zeiss model LSM 900.

### Protein modelling and structural analysis

The 3D structure of C6/36 KLC1 (AALC636_019471.R27282) was modelled with AlphaFold 3.0 [40]. Molecular graphics and analyses were performed with UCSF ChimeraX (Resource for Biocomputing, Visualization and Informatics at the University of California, San Francisco, with support from National Institutes of Health R01-GM129325 and the Office of Cyber Infrastructure and Computational Biology, National Institute of Allergy and Infectious Diseases [41]).

### Quantitative RT-PCR

Genomic DENV RNA levels were determined by qRT-PCR. Total RNA was extracted from cell monolayers washed three times with PBS, with 500 µl of Trizol reagent (Thermo Fisher, 15596018) and 100 µl of chloroform (J.T. Baker, 9180-02) added for 3 min at room temperature. The solution was centrifuged at 12,000 xg for 15 min at 4 °C. Supernatants were collected, and 250 µl of isopropanol was added, the solution incubated at room temperature for 10 min and centrifuged at 12,000 ***g*** for 10 min at 4 °C. Supernatants were discarded, and the pellet was resuspended with 1 ml of ethanol 75% and centrifuged at 7,500 ***g*** for 5 min at 4 °C. The supernatant was discarded, and the pellet was resuspended with 20 µl of nuclease-free water and treated with DNAse I (BioLabs Cat. M0303).

The qRT-PCRs were normalized with GAPDH mRNA as a housekeeping gene. For each reaction, 5 µl of qPCR SyGreen 1-Step Go Hi-ROX (PB25.32-03, PCR Biosystems), 0.5 µl of a stock of primers (10 µM) and 1 µl of RNA diluted in RNAse-free water were added. Retro-transcription was performed at 50 °C for 10 min, followed by enzyme inactivation and DNA denaturation at 95 °C for 2 min; finally, 40 cycles of 5 s at 95 °C and 30 s at 60 °C were performed on an Eco Illumina System. The threshold was adjusted with a non-infected sample and a non-template control. The results were analysed with the EcoStudy software version 5.04890.

### Quantification of secreted NS1

The amount of cell-secreted NS1 was quantified using a homemade ELISA. Plates (Corning, 3590) were coated with 100 µl well^−1^ of a rabbit polyclonal NS1 antibody (5 µg final concentration) diluted in bicarbonate buffer (0.1 M pH 9.6) overnight at 4 °C. After removing the coating solution, wells were washed four times with PBS 0.05% Tween-20 and blocked with 200 µl of blocking solution (PBS, 1% BSA) for 1 h at room temperature. After four washes with PBS 0.05% Tween-20, cell supernatants, diluted in PBS 0.05% Tween-20, 0.1% BSA, were added, and plates were incubated for 2 h at room temperature. Wells were washed four times with PBS 0.05% Tween-20 and incubated with 100 µl of 7E11 biotinylated NS1 antibody diluted 1:4,000 in dilution buffer (PBS 0.05% Tween-20, 0.1% BSA) for 2 h at room temperature. The wells were washed four times with PBS 0.05% Tween-20, and then 100 µl of streptavidin-HRP 1:8,000 was added for 1 h at room temperature. Finally, the wells were washed four times with PBS 0.05% Tween-20, and 100 µl of ABTS substrate (Sigma, A-1888) with H2O2 0.3% was incubated for 1 h. A commercial recombinant DENV NS1 protein (R&D Systems), serially diluted, was used as a standard to build the calibration curve.

### Co-immunoprecipitation

The co-immunoprecipitation assays were performed using a commercial kit (Abcam, ab206996) according to the manufacturer’s instructions. For each sample, 25 µg of NS1 antibody was incubated in 500 µg of mock or infected cell lysates harvested at 24 hpi. Controls include mock- and DENV-infected cell lysates (input) and a mock-infected eluted fraction (immunoprecipitate). Input and eluted samples were analysed by western blot using antibodies against NS1 (Abcam, ab150111) and KLC1 (ABclonal, A5552) as primary antibodies. Results were documented with a Newton Mini FX7 Edge 18.11 – SN hardware.

### Immunoelectron microscopy

C6/36 cell monolayers were washed with PBS, scraped and centrifuged at 100 x***g*** for 8 min at 4 °C. The pellet was resuspended with glutaraldehyde 0.5% and paraformaldehyde 4% and incubated at room temperature for 1 h. Cells were washed with PBS and dehydrated with increasing concentrations of ethanol (50, 70, 90 and 100%). Afterwards, cells were embedded in LR-white pure resin (London Resin Co.) and polymerized for 48 h at 4 °C in UV light. The thin sections were incubated overnight with anti-NS1 (Abcam, ab150111) and anti-KLC1 (ABclonal, A5552) antibodies at 4 °C. The next day, samples were washed with PBS and incubated for 1 h with goat anti-mouse (Ted Pella, Inc.) and goat anti-rabbit IgG (H+L) (Ted Pella, Inc.) secondary antibodies coupled with 15 and 30 nm gold particles, respectively. Finally, samples were washed with PBS and incubated with lead citrate and uranyl acetate and observed with a JEOL-JEM-01400 transmission electron microscope.

### Lipid droplets staining and counting

For quantification of LD, cells were stained with Oil Red (Matheson Coleman and Bell) for 30 min; then, the slides were washed three times with PBS-Tween for 10 min each. Finally, LD were visualized using the indirect immunofluorescence protocol described above. LD were counted with the ImageJ software [42] with the Analyze particles plug-in. The threshold and detection range were set for the control images; once these parameters were established, they were applied for the analysis of the LD in the experimental samples.

### Peptide assays

KINTAG is a peptide of 17 residues with an FITC tag coupled by an Ahx linker (pepMic Co., Ltd., Jiangsu, China) that binds to the cargo region of KLC1 [34]. To evaluate the peptide transduction effect on the cell’s viability and LD homeostasis, C6/36 cell monolayers were washed once with PBS and incubated with KINTAG peptide, diluted in MEM-FBS 5% to a final concentration of 0.1 µg µl^−1^, for 24 h at 27 °C in a 5% CO_2_ atmosphere. The effect of the KINTAG on DENV infection was evaluated by treating the cells at 2 hpi, and the infection was allowed to proceed until 24 hpi. At this time, cells were harvested and processed for further analysis.

### Statistical analysis

Results were analysed for statistical difference using GraphPad Prism version 8.0.2.

## Results

### KLC1 interacts with NS1 in DENV-infected C6/36 cells

**Fig. 1.**
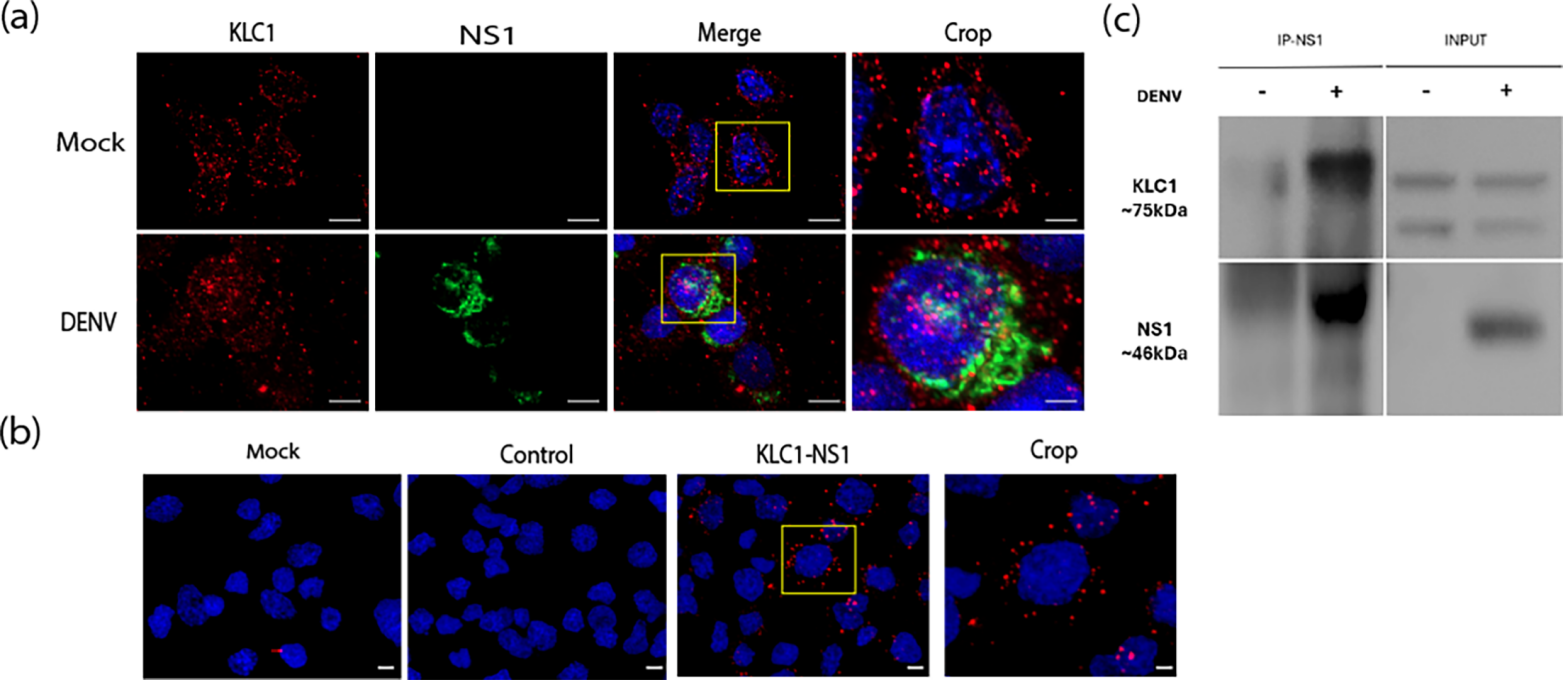
KLC1 interaction with DENV NS1 in infected C6/36 cells. (**a**) Colocalization assays of KLC1 and DENV NS1 (Pearson=0.52). (**b**) PLA of KLC1 and DENV NS1. Mock-infected cells treated with anti-KLC1 (*mock*) and infected cells with primary anti-KLC1 antibody omitted (*control*) were included as negative controls. Crop in (a) and (b); enlargement of the cells enclosed in the yellow square. (**c**) Immunoprecipitation assays. Cell lysates were immunoprecipitated with anti-NS1 antibodies bound to Sepharose beads. The presence of DENV KLC1 in the immunoprecipitant was revealed by western blot. Changes in protein migration observed in the immunoprecipitate are due to elution buffer composition and were consistently observed in experimental replicas. In all cases, mock and infected cells were harvested at 24 hpi. C6/36 average cell diameter=12.5 µm. Bar=5 µm. Representative images of at least three independent experiments are shown.

Previous evidence from our group suggested interactions between KLC1 and NS1 in infected C6/36 cells [39]. To corroborate this finding, immunofluorescence, PLA and immunoprecipitation assays were carried out in DENV-infected C6/36 fixed at 24 hpi, using mock-infected cells as controls (Fig. 1). Colocalization between KLC1 and NS1 was observed, mainly in the cytoplasm (PCC=0.52) (Fig. 1a). In addition, positive signals were observed in the cytoplasm of infected cells by PLA, while signals were not observed in the negative controls, run in parallel, indicating specific interaction between KLC1 and NS1 (Fig. 1b). Finally, pull-down of NS1 from DENV-infected cell lysates showed the presence of one KLC1 isoform in the immunoprecipitated fraction, absent in mock-infected cell lysates (Fig. 1c). All these results taken together indicate that KLC1 and DENV NS1 interact in C6/36 infected cells.

### KLC1 modelling and docking assays with NS1

**Fig. 2.**
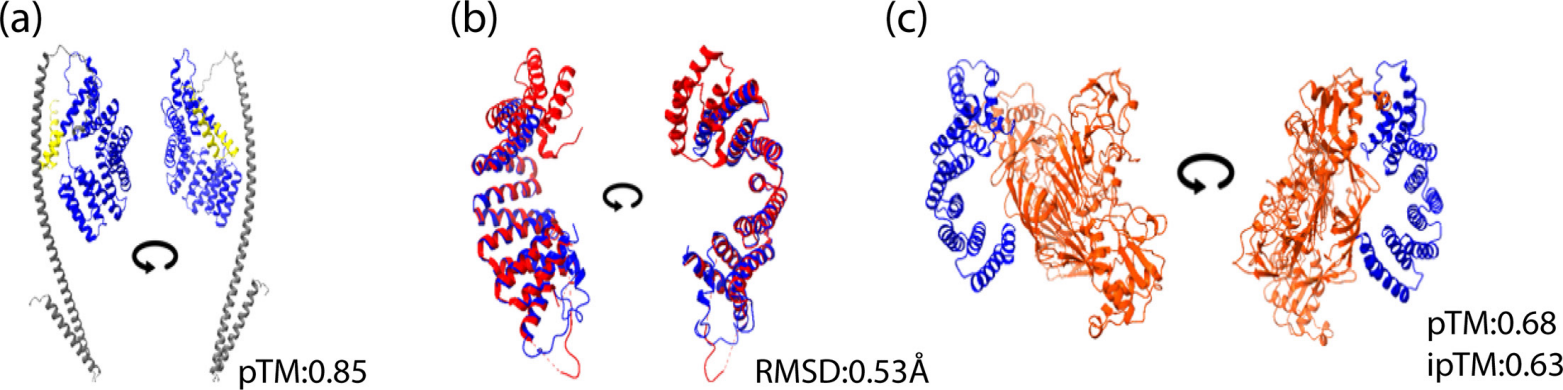
*In silico* modelling of mosquito KLC1. (**a**) AlphaFold 3.0 modelling of the KLC1 of *A. albopictus*. TRP domain shown in blue. (b) Superimposition of the TPR cargo binding domain of mosquito (blue) and human (red) KLC1. (**c**) Modelled interaction of the TPR (blue) and NS1 dimers (orange). pTM: predicted template modelling. ipTM: interface predicted template modelling. RMSD: root mean square deviation. Circular arrow=180° image rotation.

To gain insights into the KLC1 and NS1 interactions, the full KLC1 molecule was modelled via AlphaFold 3.0, since only the TPR domain crystallographic structure is available [33]. The KLC1 model obtained showed a predicted template modelling (pTM) of 0.85, indicating high confidence in the predicted structure (Fig. 2a). Moreover, the comparison of the TPR region of *Homo sapiens* with the predicted TPR region of C6/36 KLC1 showed a root mean square deviation value of 0.53 Å (Fig. 2b), indicating high structural resemblance. Finally, docking assays between the TRP domain of KLC1 and the NS1 dimer showed a possible interaction between the *β*-roll region of the NS1 dimer and the inner groove of the TPR domain with a pTM of 0.68 and an interface predicted template modelling of 0.63 (Fig. 2c).

### KLC1 colocalizes with NS1 on the surface of membrane-bound organelles

**Fig. 3.**
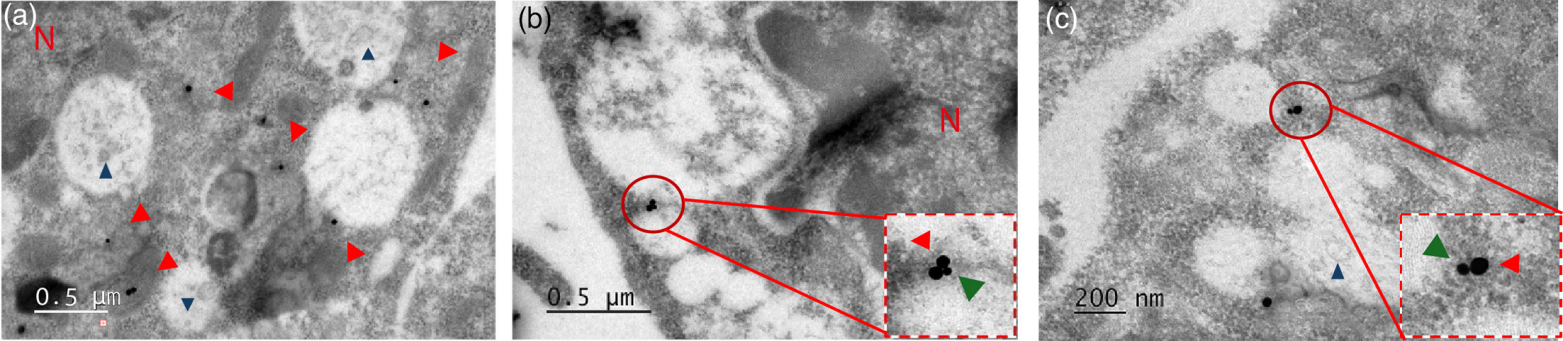
Immunoelectron microscopy of C6/36 cells infected with DENV. KLC1 was labelled with 30 nm (red arrowheads), and NS1 with 15 nm (green arrowheads) gold particles. (a) Mock-infected cells; (b) and (c) infected cells. Cells were fixed at 24 hpi. KLC1 and NS1 were found to colocalize around vacuole-like structures. N=cell nuclei. Blue arrowheads=lipid cores.

To gain further information on the interaction between KLC1 and NS1, infected cells were analysed by immunoelectron microscopy to determine the subcellular regions where these interactions take place. In mock-infected cells, the mark for KLC1 was observed on the surface of round organelles of different sizes, with ultrastructural characteristics fully compatible with LD; that is, round and electron-translucent, lacking a surrounding lipid bilayer and the presence of a lipid core [43]. In infected cells, the mark for KLC1 remained on the surface of these organelles but was accompanied by the mark of NS1 (Fig. 3). No mark for NS1 was observed in the mock-infected cells, and no significant differences in size or number were observed for LD between mock or infected cells.

These results suggest that the interaction between KLC1 and NS1 occurs on the surface of LD, a localization previously undescribed for DENV NS1, but in agreement with the capacity of both KLC1 and NS1 to interact with lipids [34,44, 45]. To corroborate NS1 interaction with LD, confocal microscopy assays were carried out. The results showed colocalization (PCC=0.34) between NS1 and LD (Fig. S1A, available in the online Supplementary Material), suggesting that a fraction of the intracellular pools of NS1 is associated with LD.

### KLC1 is overexpressed in DENV-infected C6/36 cells

**Fig. 4.**
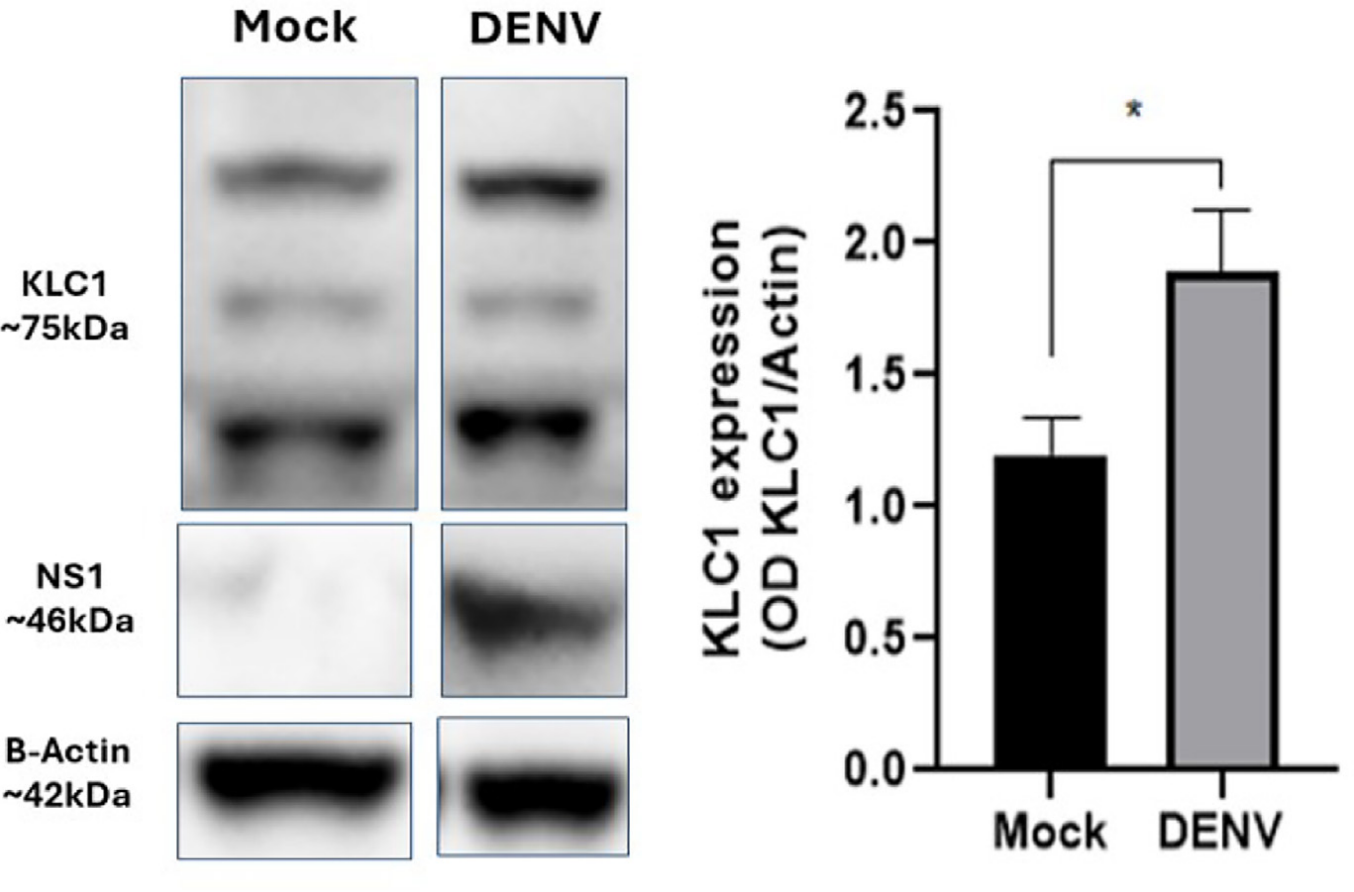
KLC1 expression levels in DENV-infected cells. Cell lysates of mock- and DENV-infected cells were analysed by western blot. Relative KLC1 protein levels are expressed as a ratio to *β*-actin used as load control. Representative images of three independent experiments are shown. *P≤0.005.

One common signature of *Orthoflavivirus* replication is the overexpression of host susceptibility cellular proteins, which aid viral replication [39,46,49]. In this sense, significantly increased levels of KLC1 were observed by western blot in infected cells harvested at 24 hpi (Fig. 4), when compared to mock-infected cells. Increments in KLC1 levels were observed starting at 6 hpi but did not reach significance until 12 hpi (data not shown). These results suggest that KLC1 expression is increased in DENV-infected mosquito cells.

### KLC1 is a susceptibility factor for DENV infection in C6/36 cells

**Fig. 5.**
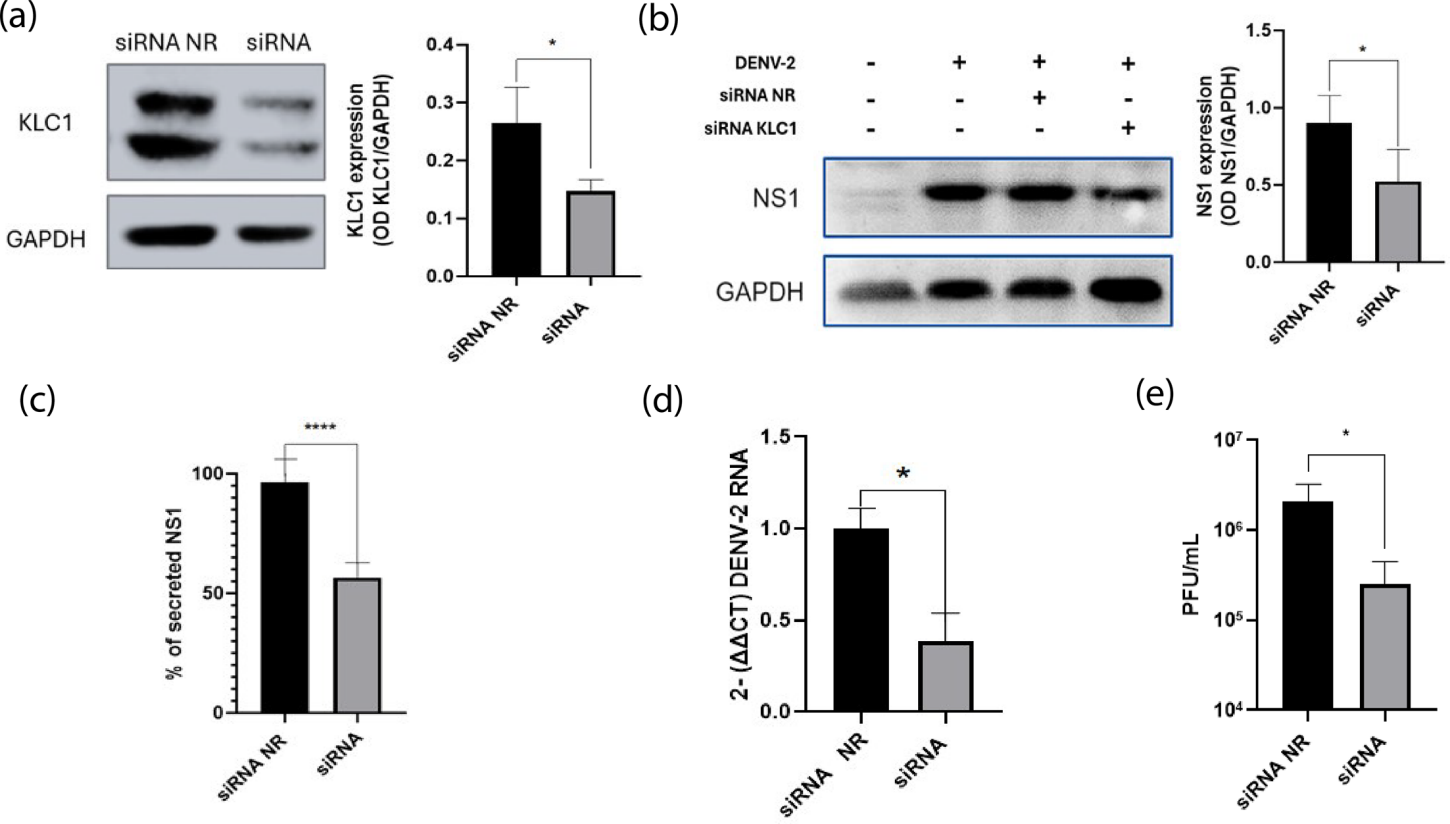
Effect on DENV replicative cycle of silencing the expression of KLC1. (**a**) Transfection of C6/36 cells with siRNA specific for KLC1 or an NR siRNA. Cells were lysed 24 hpt, and the level of protein expression was analysed by western blot. Relative KLC1 protein levels are expressed as a ratio to GAPDH. (**b**) Expression levels of NS1 in DENV-infected cells silenced or not for KLC1 and lysed 24 hpi. NS1 levels are expressed as a ratio to GAPDH. (**c**) Amount of secreted NS1 in cells silenced or not for the expression of KLC1. Secreted NS1 was measured by an in-house ELISA, expressed as a percentage of the control condition, taken as 100%. (**d**) Virus genome levels. RNA levels were determined by qRT-PCR. (**e**) Virus yield. Virus progeny was determined by plaque assay. Cells were lysed and supernatants collected at 24 hpi. *P≤0.005. ****P≤0.001. *n*≥3.

To gain insight into the participation of KLC1 in the replicative cycle of DENV, the expression of KLC1 was knocked down using siRNAs. A reduction of 50% in the expression of KLC1, as assayed by western blot, was achieved in mock-infected cells transfected with an effective siRNA-KLC1 concentration of 200 nm (Fig. 5a); a concentration proven to be non-toxic for C6/36 cells (Fig. S2A). The transfection of infected C6/36 cells with siRNA-KLC1 resulted in a ~50% reduction in the levels of the NS1 protein, measured at 24 hpi (Fig. 5b). The amount of secreted, soluble NS1 was also reduced by 50% (Fig. 5c), compared to irrelevant siRNA transfected cells, reflecting the decrease observed in the intracellular levels of NS1. In addition, viral genome synthesis and virus progeny were also evaluated. As shown in Fig. 5(d, e), a significant reduction in the relative expression of genomic RNA and in virus yield was observed in KLC1 silenced cells, in comparison with cells transfected with the irrelevant siRNA used as a control. These results suggest that KLC1 silencing affects the overall DENV replication cycle.

**Fig. 6.**
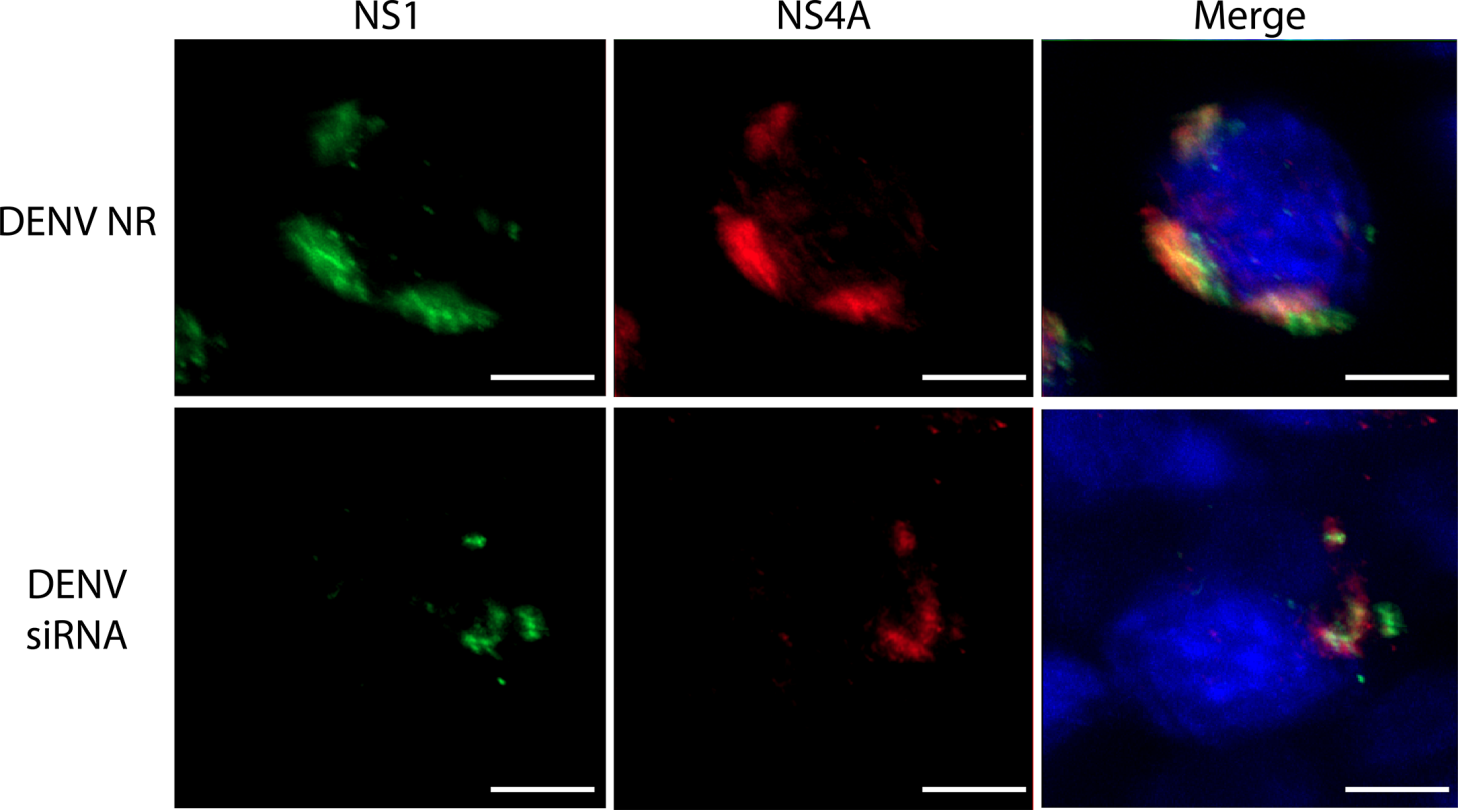
Effect on DENV RC of cells silenced for KLC1. Cells transfected with an NR siRNA. Cells transfected with a KLC1-specific siRNA. Cells were fixed at 24 hpi. Bar=5 µm. Representative images of three independent experiments are shown.

Thus, confocal microscopy assays staining for NS1 and NS4A were carried out as a measure of the integrity of the RC, given that NS4A is an integral part of these structures [21]. A reduction in both NS1 and NS4A signals was observed in infected cells silenced for KLC1 in comparison with non-related (NR) siRNA transfected cells (Fig. 6). Moreover, the colocalization between NS1 and NS4A was reduced in the KLC1 silenced cells (PCC=0.40 vs 0.31), suggesting affectation in the integrity of the RC. These results altogether revealed that KLC1 is necessary for the DENV replication cycle in mosquito cells.

### The KINTAG peptide affects DENV replicative cycle

**Fig. 7.**
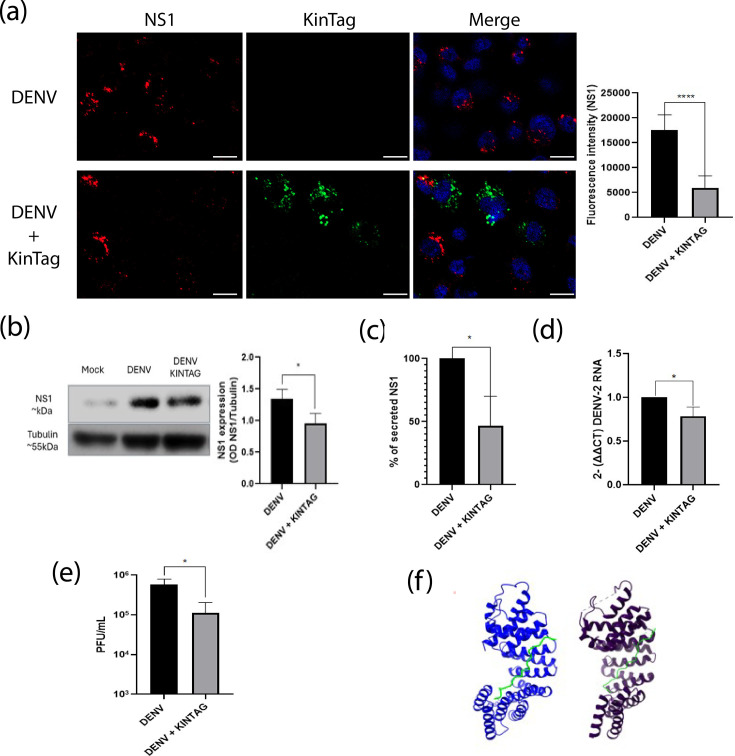
Effect of the KINTAG peptide on the replication of DENV. (**a**) Fluorescence intensity of NS1 in cells transfected with KINTAG peptide fixed at 24 hpi. (**b**) Expression levels of NS1 in DENV-infected cells with KINTAG peptide 24 hpi. NS1 levels are expressed as a ratio to tubulin. (**c**) Amount of secreted NS1 in cells with KINTAG peptide. Secreted NS1 was measured by an in-house ELISA, expressed as a percentage of the control condition, taken as 100%. (d) Virus genome levels. RNA levels were determined by qRT-PCR. (**e**) Virus yield. Virus progeny was determined by plaque assay. Cells were lysed and supernatants collected at 24 hpi. (**f**) Comparison between 6SWU crystal structure (purple) and C6/36’s KLC1 TPR and KINTAG peptide interaction predicted by AlphaFold (blue); KINTAG peptide is shown in green. Bar=10 µm. *P≤0.005. ****P≤0.001. *n*≥3.

To corroborate the results obtained in cells silenced for KLC1, infected cells were transduced with KINTAG, a 17-residue peptide with high affinity to the TPR region of KLC1 of mice [33]. The rationale for KINTAG use is to interfere with the ability of the TPR region of KLC1 to bind cargo proteins. The peptide was used at a final concentration of 0.1 µg µl^−1^, the highest non-lethal concentration denoted for C6/36 cells (Fig. S2B). Fig. 7(a) shows the cell internalization of KINTAG evaluated at 24 h after exposure by confocal microscopy. Infected cells treated with KINTAG 2 hpi showed significantly reduced levels of NS1 protein synthesis and secretion, and also significantly reduced viral genome and virus yield levels at 24 hpi (Fig. 7b–e). These results corroborate the results obtained using siRNAs and support the notion that a functional KLC1 is a necessary factor for the DENV replication cycle. Finally, docking assays indicated that, as expected, KINTAG has the potential to interact with the TPR region of C6/36 KLC1, showing that the association between the peptide and the inner groove of the TPR region is similar to the interaction observed with the TRP crystal 6SWU [33] (Fig. 7f). In agreement with the docking assay results, transfection of mock-infected cells with KINTAG at 0.1 µg µl^−1^ showed colocalization of the peptide with KLC1 (PCC=0.66), suggesting the formation of KLC1–KINTAG complexes (Fig. S1C).

### KLC1 is involved in LD homeostasis

**Fig. 8.**
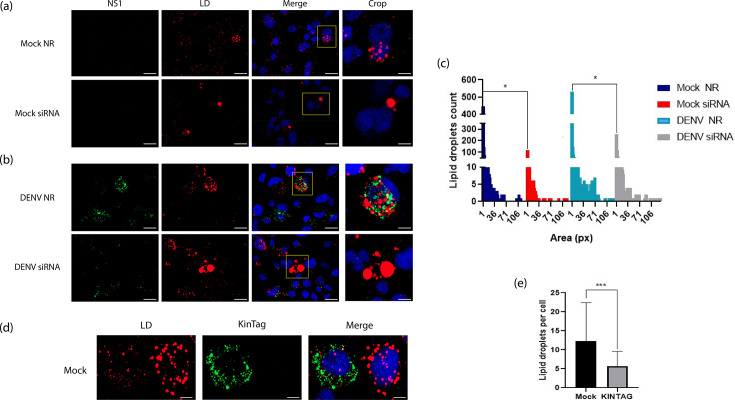
Effect of KLC1 silencing or function disruption on LD homeostasis. (**a**) Mock-infected cells silenced (siRNA) or not (NR) for KLC1 expression. (**b**) DENV-infected cells silenced or not for KLC1 expression. Cells were fixed at 24 hpi. (**c**) LD count in mock and infected cells, silenced or not for KLC1. The graph represents the LD count and area measured in pixels of 130 cells for each experimental condition. (**d**) Mock-infected cells transfected with KINTAG, fixed at 24 hpt. (**e**) LD count in KINTAG transfected cells. LD were stained with oil red-O. Bar=10 µm. Representative images of three independent experiments are shown. *P≤0.005.

The KMC is known to be involved in the trafficking and mobilization of LD, organelles that are required for the DENV replication cycle [27,50]. Interestingly, major changes in the number or area of LD were observed in either mock or infected C6/36 cells treated with siRNA-KLC1 (Fig. 8a, b). In cells with KLC1 synthesis diminished, the number of LD per cell was significantly reduced, while the mean area of LD increased (Fig. 8c), suggesting that either coalescence of LD is taking place or proper dispersion is affected. Interestingly, infected cells with altered LD patterns also presented a marked reduction in NS1 fluorescence signal (Fig. 8b). To further evaluate a role for KLC1 in LD homeostasis, mock-infected cells were transfected with KINTAG. A significant reduction in the number of LD per cell was also observed in the treated cells (Fig. 8d, e). In agreement with the literature [35], these results indicate a role for KLC1 in the homeostasis of LD, thus hinting at a mechanism for the effects of KLC1 silencing or function alterations on the DENV replication cycle.

## Discussion

The effective control of dengue will require a better understanding of the interactions between the DENV and the mosquito vector. Yet, aspects like virus–cell interactions or virus–host protein interactions are better understood for the mammalian host than for the vector mosquito, a knowledge gap that needs to be closed. An interactome generated using a proximity biotinylation system and mass spectrometry revealed KLC1 as part of the interactome of NS1 in C6/36 cells [39]. Interestingly, KLC1 was not detected in a previous DENV NS1 interactome based on three different mammalian cell lines [51]. These dissimilar observations suggested that KLC1 may be a susceptibility factor for the DENV replicative cycle in mosquitoes. In this work, the interaction between DENV NS1 and KLC1 in C6/36 cells was demonstrated by three different specific assays, thus corroborating our previous identification of KLC1 as part of the interactome map of NS1 in C6/36 cells [39].

A role of KLC1 in the anterograde transport of viral protein cargos has been proposed in the context of several DNA virus infections [34,36, 52]. Both KLC1 and NS1 have the capacity to interact with lipid membranes and induce their bending [34,44, 45]. KLC1 is a protein proposed to interact with membrane-bound organelles by its C-terminal domain and to interact with cargos through the TPR domain located in the cytoplasm; meanwhile, intracellular NS1 is mainly located in the ER luminal side [34,44, 45]. Thus, the interaction between KLC1 and NS1 reported in this study could happen through ER or other internal membranes. Alternatively, NS1 can also interact directly in the cytoplasm with the TPR region of KLC1, making NS1 a cargo protein. This possibility is supported by the colocalization of KLC1 and NS1 on the surface of LD observed by immunoelectron microscopy and confocal microscopy and the docking assays between NS1 and the C6/36 KLC1 TPR region, showing a likely interaction between the TPR domain of KLC1 and the *β*-roll domain of NS1 (Fig. 2c). Of note, evidence suggesting the presence of a fraction of NS1 in the cell cytoplasm is indirect but has been accumulating in recent years [39,51, 53].

Regardless of the KLC1 cargo condition of NS1, our results indicate that KLC1 is a susceptibility factor for DENV in mosquito cells. RNA replication, protein synthesis, NS1 secretion and virus progeny were all processes significantly affected in infected cells silenced for the expression of KLC1 or transduced with a high-affinity peptide for the TRP domain. In addition, the levels of KLC1 were found to significantly increase at 24 hpi, in agreement with previous results obtained measuring KLC1 RNA levels in DENV-infected C6/36 cells [54]; increased expression of cell susceptibility factors has been frequently observed in *Orthoflavivirus*-infected cells [46,48]. A plausible explanation for the overall ‘up-river’ effect observed in the absence of KCL1 is that the KMC participates in the reorganization and formation of the RO in the infected cells. Indeed, in the absence of KLC1, the colocalization coefficient index between NS1 and NS4A was significantly reduced, suggesting a disruption of the RC. These results are in line with a series of studies that address the hijacking of the cytoskeleton and the cytoplasmic streaming system by Orthoflaviviruses [55,58]. Noteworthy, KLC1 has been studied in the context of other viral infections [38,52] and was proposed to be involved in the anterograde transport of viral cargoes, positioning it as a frequent target of viral effectors.

The presence of a fraction of the intracellular NS1 pool associated with LD and the interaction with KLC1 on their surface suggests mechanistic explanations for the KLC1–NS1 interaction [59,61]. Colocalization between the microtubules and the NS4A protein and between KLC1 and the NS3 protein was observed in infected cells (Fig. S1B, D), suggesting a connection between KMC and the DENV RO, as observed in other Orthoflaviviruses [58,62]. The RO and the LD alike originate from the membranes of the ER [14,63, 64]; in consequence, the NS1–KLC1 complex may act to promote lipid mobilization and the formation of LD from the ER membranes. Alternatively, NS1 could tether by its interaction with KLC1, the RO to the KMC, and close to LD, thus enhancing lipid flux to favour the channelling of triglycerides and cholesterol into these structures [65,68]. Several subunits of the KMC, including KLC1, have been reported to be involved in the mobilization of LD through the microtubules [30,35]. In support, silencing KLC1 expression or interfering with KLC1 function resulted in altered LD distribution (fewer and enlarged LD) and an overall impact on the DENV replicative cycle. Moreover, it has been reported that silencing other members of the KMC like KIF5-B [31] also causes alterations in LD homeostasis, similar to the ones observed in this study. The direct association of NS1 with LD has not been reported before, and as it has a different pattern to the one reported for the DENV or HCV C proteins [27,28], it is not surprising given the amphipathic nature of NS1 [45]. It is clear that the significance of the KLC1 and NS1 interaction needs further investigation, but the possibility of uncovering new functions for NS1, derived from its association with KLC1 and LD, is intriguing.

In summary, this study reveals a role for KLC1 as a susceptibility factor for DENV replication in mosquito cells. The participation of KLC1 as part of the KMC in the lipid hijacking processes in mosquito cells by DENV, particularly in relation to LD, appears to be one of the functions that KLC1 fulfils during the replication cycle. Nonetheless, other KLC1 functions need to be evaluated in relation to the DENV replicative cycle. Also, the need for KLC1 in DENV infection of vertebrate cells needs to be evaluated, even though KLC1 did not show up in an interactome of DENV NS1 derived from three vertebrate cell lines [55]. Despite the limitations, this work contributed to the understanding of virus–cell interactions and points out KLC1 as a new potential antiviral target for Orthoflaviviruses.

## Supplementary material

10.1099/jgv.0.002132 Uncited Supplementary Material 1.

## Abbreviations

DENV: dengue virus
ER: endoplasmic reticulum
hpi: hours post-infection
hpt: hours post transfection
KLC1: kinesin light chain 1
KMC: kinesin motor complex
LD: lipid droplets
MEM: minimum essential medium
NR: non-related
NS: non-structural
PCC: Pearson correlation coefficient
PLA: proximity ligation assays
pTM: predicted template modelling
RC: replication complexes
RO: replication organelles
TPR: tetratricopeptide repeat
