# Supplementary material for: Kinesin light chain 1 interacts with NS1 and is a susceptibility factor for dengue virus infection in mosquito cells
Source: J Gen Virol. 2025 Jul 16;106(7):002132. doi: 10.1099/jgv.0.002132 (PMC12266351; doi:10.1099/jgv.0.002132)
Supplement: Uncited Supplementary Material 1. [file jgv-106-02132-s001.pdf]

Supplemental figure 1

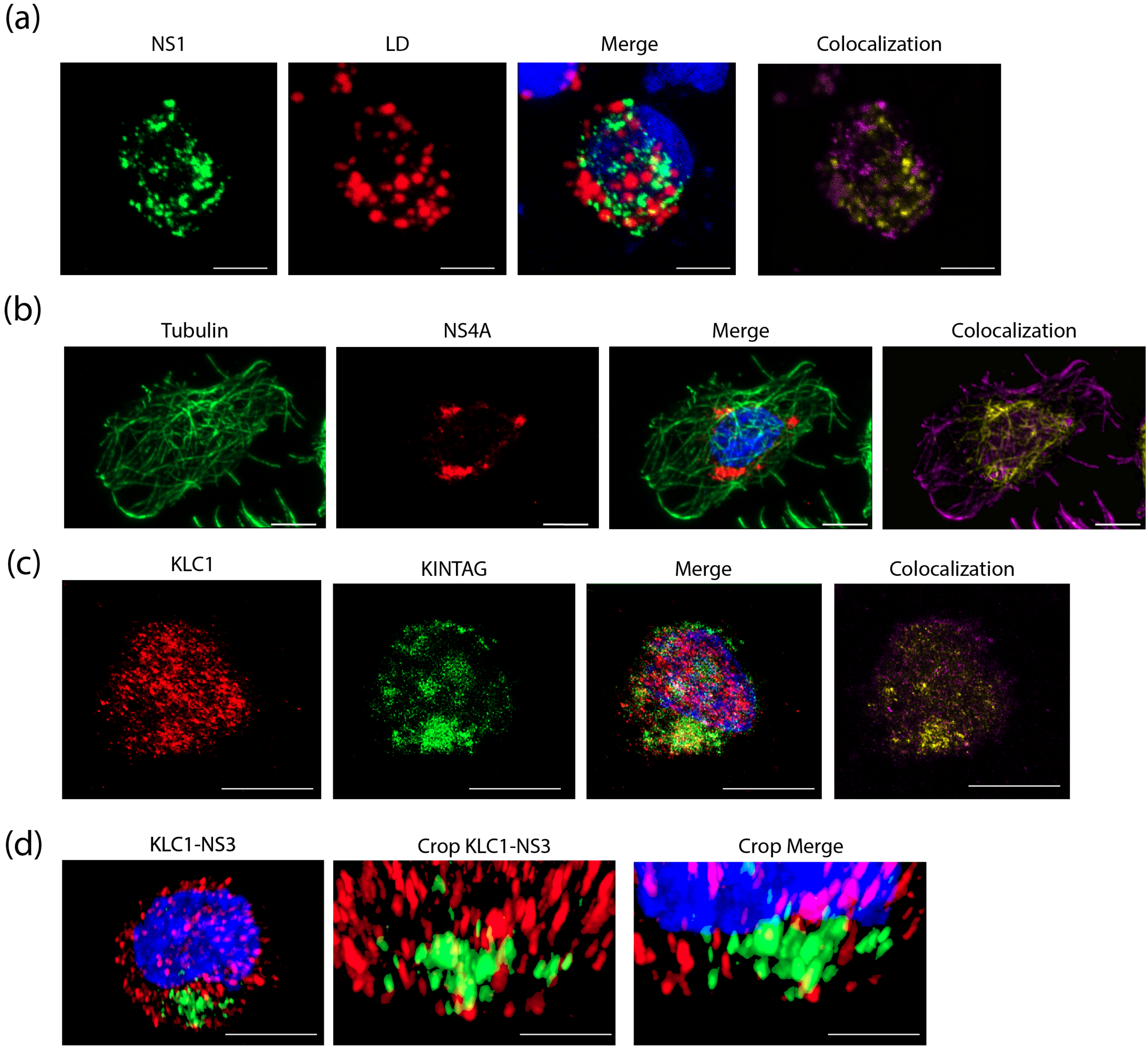

Supplemental figure 1. A) Colocalization between LD and NS1; Pearson correlation coefficient=0.34 B) Colocalization between tubulin and NS4A; Pearson correlation coefficient=0.72. C) Colocalization between KINTAG and KLC1; Pearson correlation coefficient=0.66. D) Colocalization between KLC1 and NS3; Pearson correlation coefficient=0.75. In A) B) and D) DENV infected C6/36 cells were fixed at 24hpi (n=3). In C) mock-infected cells were used (n=2). Colocalization areas are shown in yellow and anti-colocalization areas are shown in purple. Bar= 5 μm.

Supplemental figure 2

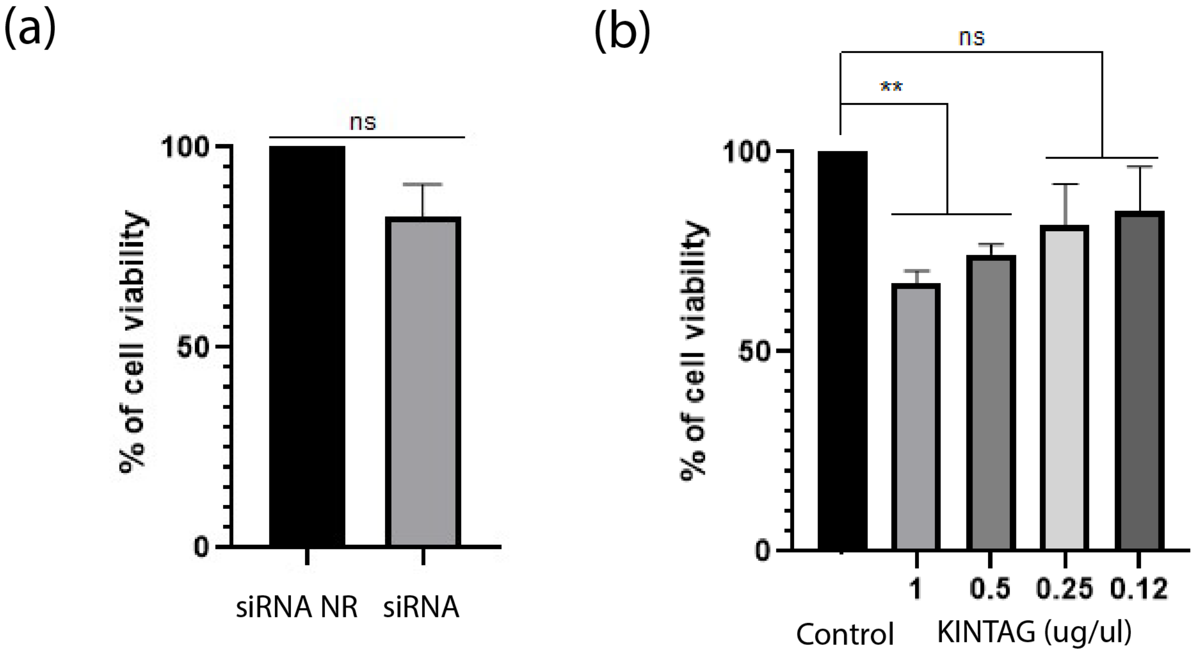

Supplemental figure 2. Effect of KLC1 silencing and KINTAG peptide transduction on C6/36 cells viability. A) KLC1 siRNA 200nM. B) KINTAG peptide in concentrations ranging from 0.12µg/µl to 1.0 µg/µl. MTS assay was performed 24 hpt. Results are the means of 3 independent experiments. ** $p \leq 0.01$.
